# Supplementary material for: Upregulated Matrisomal Proteins and Extracellular Matrix Mechanosignaling Underlie Obesity-Associated Promotion of Pancreatic Ductal Adenocarcinoma
Source: Cancers (Basel). 2024 Apr 21;16(8):1593. doi: 10.3390/cancers16081593 (PMC11048773; doi:10.3390/cancers16081593)
Supplement: Supplementary file 1 [file cancers-16-01593-s001.zip › Waldron Supplementary Figures.pdf]

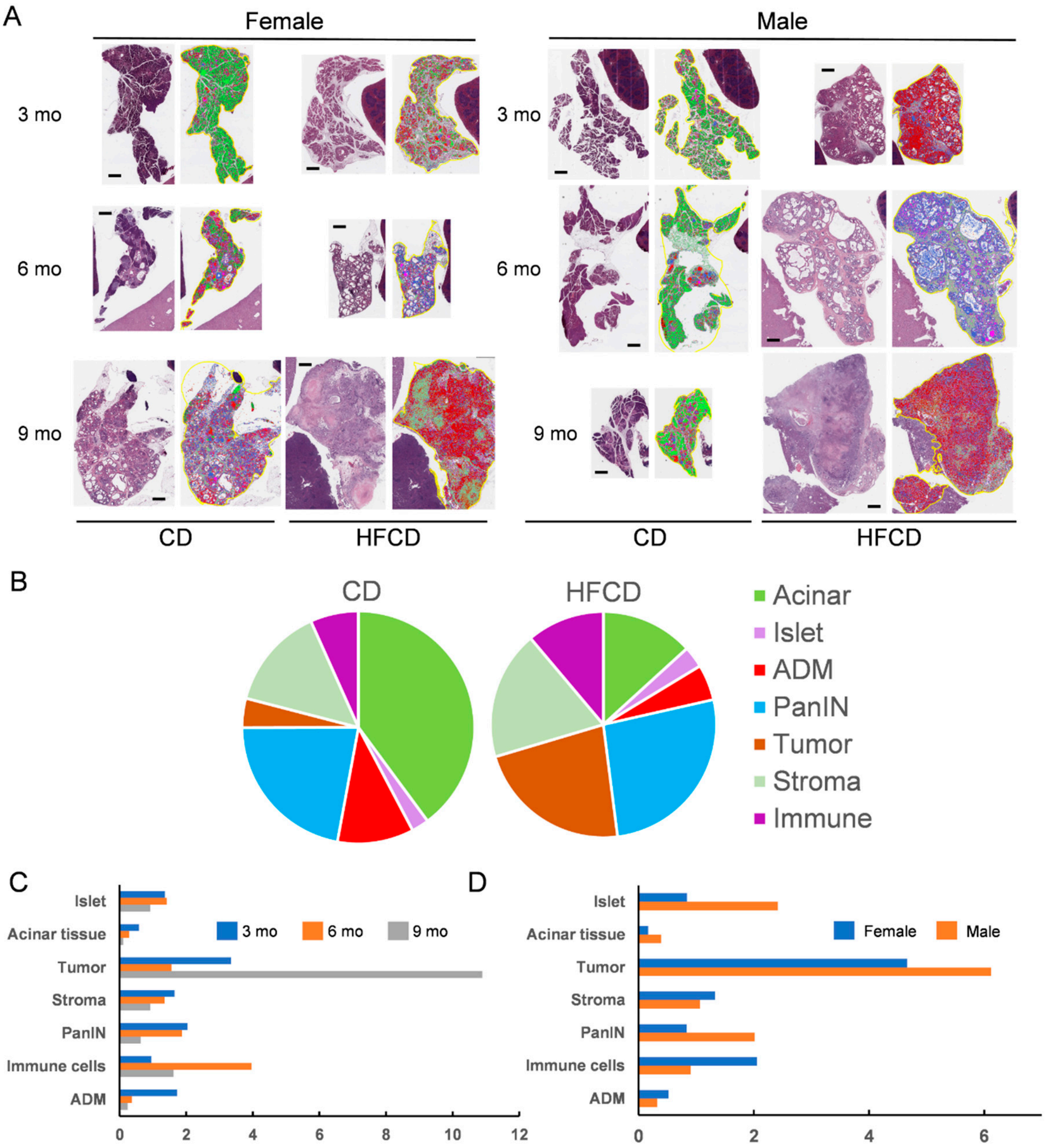

Supplementary Figure 1. DIO altered cellular phenotypes associated with accelerated PDAC in KC mice.

Supplementary Figure S1. Quantitation of predominant cell types accompanying DIO-induced PDAC progression in KC mice by QuPath classification of H&E stained images. **A**, Representative female and male H&E histology and all cell types analyzed. *Scale bars, 500 μm*. **B**, Quantitation of relative cell type composition of the HFCD- versus CD-fed KC mice tissues analyzed in A. **C**, DIO-induced cell type distribution across distinct time points. **D**, DIO-induced cell type distribution in females and males.

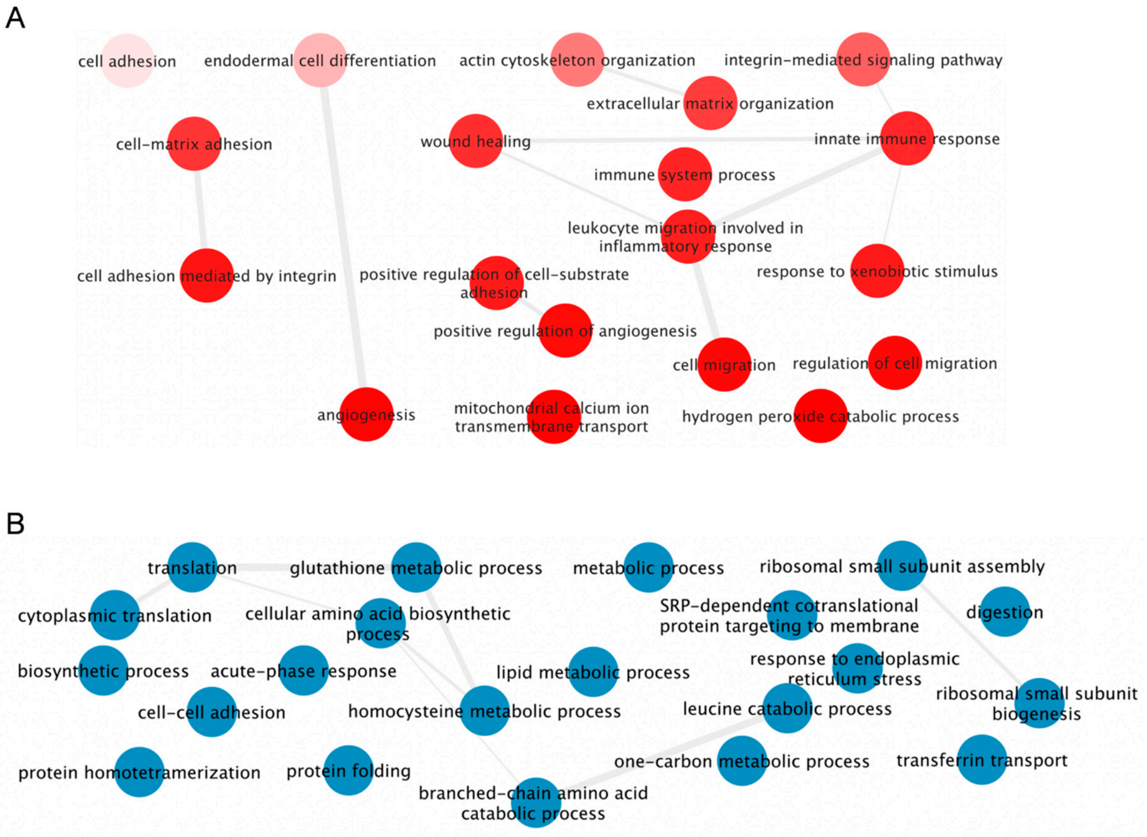

Supplementary Figure 2. DIO-associated increased and decreased proteins in KC mice.

Supplementary Figure S2. Maps of significant Biological Processes associated with DIO-induced proteins upregulated (**A**) and downregulated (**B**) in KC mice. Biological Processes GO terms analyzed by DAVID were reorganized in interconnected Revigo maps. *Red, upregulated; Blue, downregulated*.

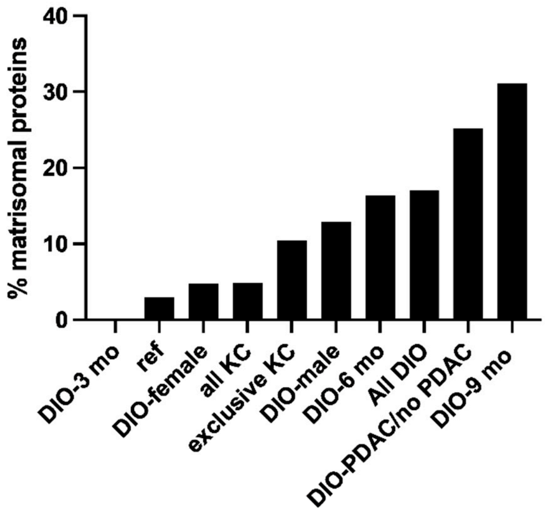

Supplementary Figure 3. Matrisomal protein enrichment in proteomic data from the KC mice.

Supplementary Figure S3. Matrisomal protein enrichment in proteomic data from the KC mice. The degree of matrisomal protein enrichment was calculated in each group of proteins elevated by DIO at least 1.5-fold in their respective subgroups, expressed as the percent matrisomal among the proteins represented. We included the matrisomal enrichment of a published[68] mouse pancreas proteome of >6000 proteins (not shown) that gave rise to a reference (**ref**) value. Comparison of our KC mice proteins with the reference proteome provided both **all KC** and **exclusive KC** groups.

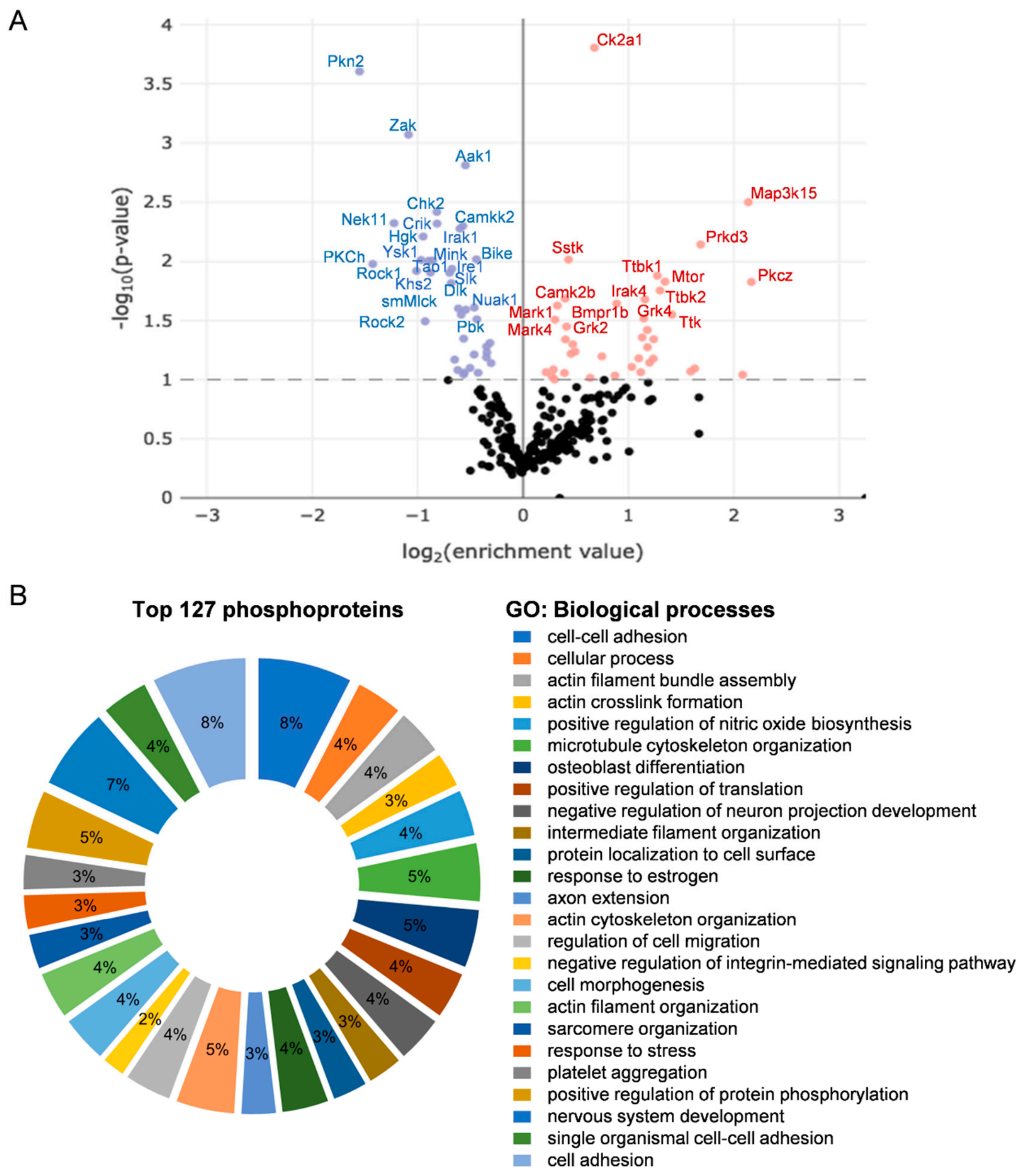

Supplementary Figure 4.

Supplementary Figure S4. Summarized bioinformatic analysis of phosphopeptide results. **A**, Volcano plot of up- or down-regulated protein kinase activities during DIO-induced PDAC progression in KC mice predicted from phosphopeptide data using the Enrichment analysis feature of the Kinase Library web tool at PhosphoSitePlus. Data summarize expression of phosphoproteins up- and down-regulated by DIO in males and females at all time points, normalized by total expression levels of the same proteins. **B**, Biological processes from DAVID analysis of the top 127 proteins with DIO-induced phosphorylation increases during PDAC development.

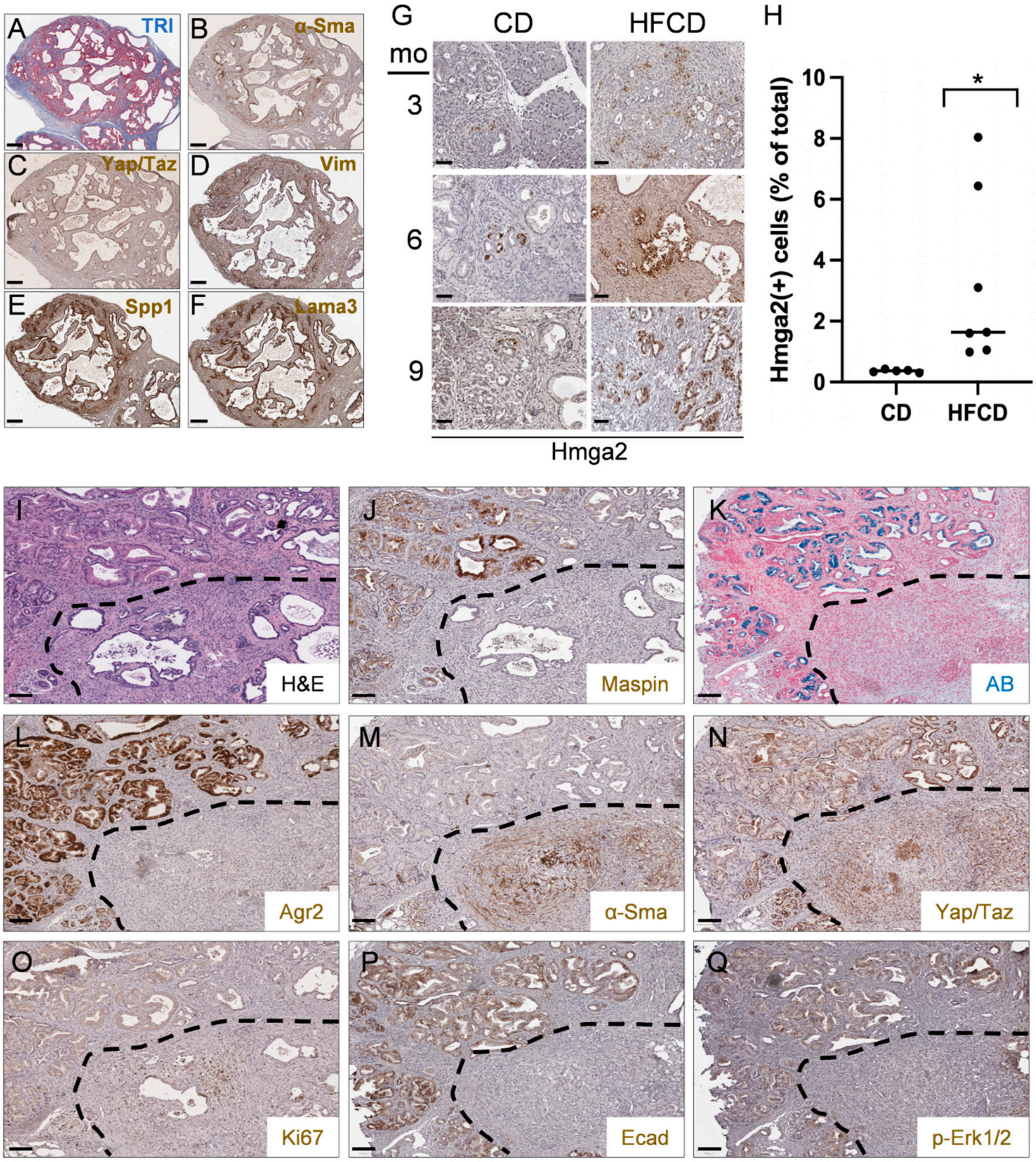

Supplementary Figure 5.

Supplementary Figure S5. **A-F**, A section of KC pancreas transforming under DIO is punctuated by AFL. **A**, Trichrome (TRI), **B**, α-Sma, **C**, Yap/Taz, **D**, Vim, **E**, Spp1, and **F**, Lama3 were stained. Groups of disorganized epithelioid cells staining negatively for Vim, modestly for Spp1 and intensely for Lama3 were interspersed with thick bands of Trichrome+ collagen and surrounded by nests of α-Sma+ myCAF. The Lama3+ cells, and stromal myCAF stain positively for Yap/Taz. *Scale bar, 200 μm*. **G**, HMGA2 staining

in 3 mo, 6 mo, and 9 mo KC mice fed CD or HFCD. *Scale bars, 50 μm*. **H**, quantitation of control (n=5) and DIO (n=7) Hmga2-stained tissue segments. **I-Q**, Pancreas from HFCD-fed KC mice stained for I, H&E or differential IHC (J-Q). Pictures show differential staining of two adjacent regions (separated by a dotted line in the panels) within a single tissue segment. In the upper PanIN enriched region, cells lining PanIN structures were positive for **J**, Maspin; **K**, Alcian Blue (AB); **L**, Agr2; **M**, α-Sma, mostly associated with blood vessels; **N**, Yap-Taz; **O**, Ki67, a marker of proliferating cells; **P**, residual Ecad, and **Q**, p-Erk. The lower region within each picture shows an emerging mesenchymal tumor with loss of lobular organization and containing alternative flat lesions, a histological signature we found associated with advanced DIO tumors. In this lower region, cells were negative for most of the markers except for abundant α-Sma+ CAF in (M), Yap/Taz in (N), Ki67+ nuclei in (O), and few p-Erk+ cells (Q). *Scale bar, 100 μm.*

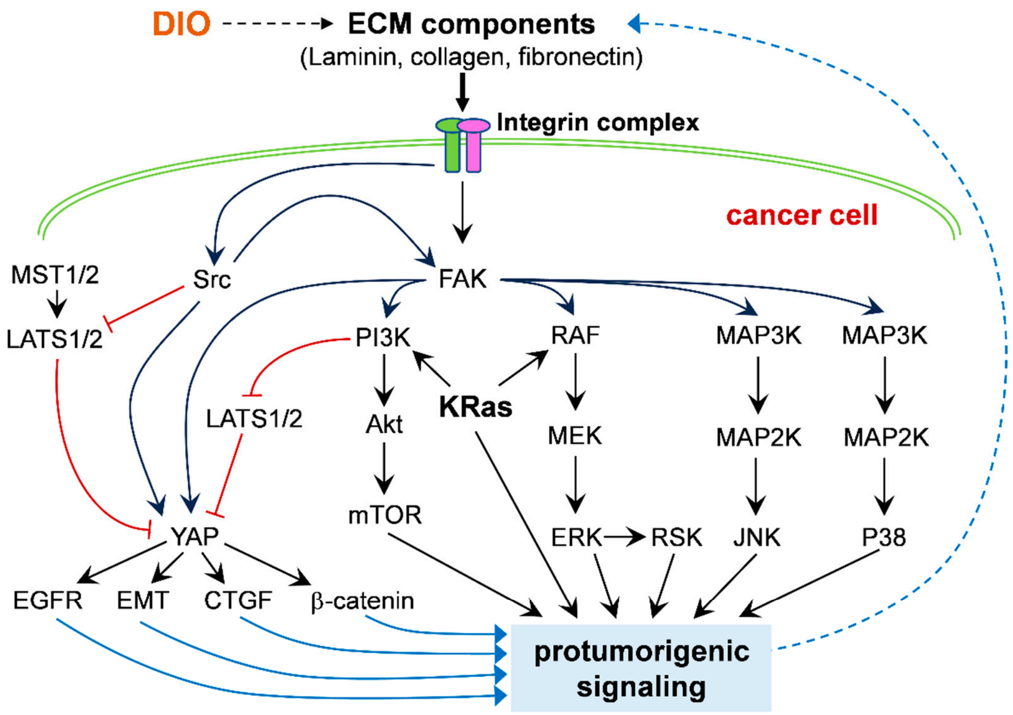

Supplementary Figure 6.

Supplementary Figure S6. Diagram of signal transduction pathways induced during DIO promotion of PDAC. DIO induces matrisomal proteins which bind integrin complexes and initiate intracellular actin cytoskeleton remodeling and focal adhesion turnover, processes associated with cell motility and invasion. Focal adhesion kinases activated via small monomeric Src family tyrosine kinases and autophosphorylation further signal via multiple pathways including MAPK and Yap/Taz to initiate protumorigenic signaling and positive feedback loops involving additional matrisomal proteins.
